# Synthesis of Sulfur@g-C_3_N_4_ and CuS@g-C_3_N_4_ Catalysts for Hydrogen Production from Sodium Borohydride

**DOI:** 10.3390/ma16124218

**Published:** 2023-06-07

**Authors:** Khulaif Alshammari, Turki Alotaibi, Majed Alshammari, Sultan Alhassan, Alhulw H. Alshammari, Taha Abdel Mohaymen Taha

**Affiliations:** Physics Department, College of Science, Jouf University, Sakaka P.O. Box 2014, Saudi Arabia; tbotaibi@ju.edu.sa (T.A.); malshammary@ju.edu.sa (M.A.); ssalhassan@ju.edu.sa (S.A.); ahalshammari@ju.edu.sa (A.H.A.); themaida@ju.edu.sa (T.A.M.T.)

**Keywords:** CuS@g-C_3_N_4_, nanosheet, optical bandgap, hydrogen generation, activation energy

## Abstract

In this work, the S@g-C_3_N_4_ and CuS@g-C_3_N_4_ catalysts were prepared via the polycondensation process. The structural properties of these samples were completed on XRD, FTIR and ESEM techniques. The XRD pattern of S@g-C_3_N_4_ presents a sharp peak at 27.2° and a weak peak at 13.01° and the reflections of CuS belong to the hexagonal phase. The interplanar distance decreased from 0.328 to 0.319 nm that facilitate charge carrier separation and promoting H_2_ generation. FTIR data revealed the structural change according to absorption bands of g-C_3_N_4_. ESEM images of S@g-C_3_N_4_ exhibited the described layered sheet structure for g-C_3_N_4_ materials and CuS@g-C_3_N_4_ demonstrated that the sheet materials were fragmented throughout the growth process. The data of BET revealed a higher surface area (55 m^2^/g) for the CuS-g-C_3_N_4_ nanosheet. The UV–vis absorption spectrum of S@g-C_3_N_4_ showed a strong peak at 322 nm, which weakened after the growth of CuS at g-C_3_N_4_. The PL emission data showed a peak at 441 nm, which correlated with electron–hole pair recombination. The data of hydrogen evolution showed improved performance for the CuS@g-C_3_N_4_ catalyst (5227 mL/g·min). Moreover, the activation energy was determined for S@g-C_3_N_4_ and CuS@g-C_3_N_4_, which showed a lowering from 47.33 ± 0.02 to 41.15 ± 0.02 KJ/mol.

## 1. Introduction

Hydrogen is an important and versatile source of energy. Hydrogen has huge potential to drive the global energy transition as a clean fuel even though it is the smallest molecule in the universe [1,2]. Different procedures may be used to extract hydrogen from a variety of sources, both renewable and nonrenewable. It is a flammable gas inside engines, and it can also be employed in a vehicle fuel cell, electricity production, or heat generation. For all these purposes, hydrogen can replace fossil fuels without radiating CO_2_ gas. In the same context, hydrogen is a neutral carrier of energy, such as electricity, but it decarbonizes non-electrified sectors, such as heavy industry, long-distance transportation, or periodic storage [3,4,5]. Increasing the rate of hydrogen production contributes to reducing costs and maximizing utilization. Therefore, work has been in full swing to develop catalysts of various materials to enhance hydrogen production. A variety of developed catalysts have been used to produce hydrogen for the benefit of society and as an alternative energy source.

Carbon nitrides, which are inexpensive and environmentally friendly materials, have recently proven to be a potential option for catalysts. Carbon nitrides and carbon nitride derivatives are promising catalysts. Carbon nitrides are substances with nitrogen and carbon serving as the backbone-building constituents [6]. The class of mostly planar structures known as graphitic carbon nitrides (g-C_3_N_4_) is generated from the parent binary molecule. Polymerized Tris-s-triazine layers are used to make bulk polymeric g-C_3_N_4_. Melamine, urea, thiourea, or a mixture of these compounds are heated to between 500 and 600 °C to produce g-C_3_N_4_ [7,8]. The study of carbon nitrides aims to comprehend the characteristics of these compounds, particularly their catalytic performance. A common explanation for the catalytic activity of catalysts is the presence of morphological defects such as vacancies, steps, corners, and edges [9]. The band gap and surface chemistry of the material can vary because of changes in the final characteristic crystal structure, which can be affected by the temperature of the pyrolysis process. In addition to being plentiful and simple to make using pyrolysis procedures, g-C_3_N_4_ is also interesting since it is non-toxic, very stable, and has no metals. Bulk samples suffer from small surface areas, average optical absorption, fast charge recombination, moderate redox ability, and limited charge carrier mobility [10,11,12]. Many strategies have been completed to improve g-C_3_N_4_ such as metal nanoparticle decoration, non-metal doping, nano-structuring and exfoliation methods, heterojunctions with other catalysts, and protonation of the surface [13,14,15].

Intrinsic doping with different non-metals has been found to be effective for H_2_ evolution, lowering the bandgap, improving catalyst surface area, and reducing non-radiative recombination by forming midgap states [16,17]. Meanwhile, exfoliation methods for g-C_3_N_4_ have concentrated on lowering layer thickness to counteract the consequences of poor charge transfer due to the minor effects of surface area. The ability to electrostatic self-assemble composite structures with negatively surface-charged materials to function as cocatalysts is one of the main benefits of positively charged g-C_3_N_4_ material [18,19]. A common method for defect control involves changing the number of vacancies in order to improve the material’s photocatalytic H_2_ evolution rate, nonlinear optical characteristics, and other physical and chemical features. Accordingly, different S [20], P [21], B [22] and TiO_2_ [23] integrated g-C_3_N_4_ were employed for catalytic hydrogen evolution. The presence of doping S in the matrix of g-C_3_N_4_ leads to the reduction of their band gap, induced charge rearrangement and improved the electron–hole separation. The direct control of the g-C_3_N_4_ band structure and the improvement of its optical absorption capability are two benefits of the sulfur doping technique. The visible light response photocatalytic performance of g-C_3_N_4_ was enhanced by using phosphor-doped graphitic carbon nitride as a catalyst. In order to improve photocatalysis, the interfacial heterojunction between g-C_3_N_4_ and TiO_2_ can offer a reliable channel for the transfer of charge. The integration of TiO_2_ into g-C_3_N_4_ delayed the electron–hole recombination and thus improved the catalytic performance.

Copper sulfide (CuS), a chalcogenide with special properties, has been proposed as a viable candidate for a variety of applications [24]. CuS is a low-cost semiconductor with high abundance and lower toxicity [25]. In stark contrast to bulk material, CuS nanomaterials have exceptional physical, chemical, structural, and surface features [26]. Solid-state reactivity of elements [27], solid-state metathesis [28], and self-propagating high-temperature synthesis [29] were used to produce copper sulfide. Meanwhile, CuS structures can be prepared via traditional chemical procedures such as the sol–gel method [30], hydrothermal [31] and solvothermal synthesis [32]. The crystal structure of CuS is typically hexagonal with a space group of P63/mmc [33]. Light emission, charge transport, photocatalysis, and thermal diffusion investigations have all made extensive use of p-type CuS semiconductors [34]. Band gaps for CuS microspheres are 2.08 eV, for CuS nanotubes are 2.06 eV, for CuS nanoflakes are 2.16 eV, and for CuS nanoparticles are 1.88 eV [35]. Therefore, CuS possesses high absorption of solar energy [36] and improved nonlinear optical properties [37]. Moreover, the electronic structure of CuS allows the useful application of these materials in photocatalysis [38].

The synthesis of metal oxide/g-C_3_N_4_ nanocomposites can be conducted through various methods such as the sonomechanical, hydrothermal, solvothermal and polycondensation synthesis methods. The sonomechanical synthesis is the most common method for the metal oxide and g-C_3_N_4_ nanosheet. This method was used to synthesize the molybdenum (MoO_3_) and g-C_3_N_4_ ultrathin sheet [39]. The disadvantage of this synthesis process is attributed to its weak interaction which may lead to dissociation after some catalytic cycles. Hydrothermal synthesis is a well-known method for nanomaterials preparation. It is a solution-based reaction where it can be carried out in a wide range of temperatures [40]. Hydrothermal synthesis has been previously used for the synthesis of CuS/g-C_3_N_4_ composites [41]. These hydrothermal preparation methods have some disadvantages such as a lack of recycling and regenerating the catalysts [42].

The solvothermal method has also been widely used for the synthesis of g-C_3_N_4_ when precursor and solvent are placed in an autoclave under a mild temperature. In this case, templates are helpful to control the morphology [43]. The solvothermal synthesis process has some disadvantages such as its synthesis process requires multi-steps in comparison with polycondensation. However, the polycondensation and solvothermal synthesis methods have been considered as low energy consumption and low-cost processes [44]. These synthesis methods require templates to prepare nanosheets with certain shapes such as nanotubes. Polycondensation is facile and common synthesis process which includes cost-effective nitrogen-rich precursors [45]. In the literature, the polycondensation process has been utilised for the NiS-g-C_3_N_4_ nanocomposites synthesis [46].

The general purpose of this study is to investigate alternative ways for tailoring the optical, structural, and catalytic characteristics of S@g-C_3_N4 and CuS@g-C_3_N_4_ nanocomposites. The used one-pot method is desirable since it is facile and not costly. The structural properties of these samples were completed on XRD, FTIR and ESEM techniques. The optical bandgap and photoluminescence analysis for these nanostructures will be investigated. Finally, the catalytic performance for hydrogen generation from NaBH_4_ will be analysed for S@g-C_3_N4 and CuS@g-C_3_N_4_.

## 2. Experimental

The polycondensation route was used to synthesize S@g-C_3_N_4_ via the thermal decomposition of thiourea in air. A 150 mL porcelain crucible with a cover was then filled with 12.5 g of thiourea that had been finely ground in an agate mortar. The crucible was heated to 550 °C in an air environment at a ramp rate of 3.0 °C/min, kept at that temperature for 2 h, and then cooled to room temperature. CuS@g-C_3_N_4_ nanocomposite was prepared via grinding of 12.5 g thiourea and 1.0 g CuCl_2_.6H_2_O by agate mortar for 30 min. The mixture was transferred to a porcelain crucible that was inserted into a muffle furnace. The heating process was completed at 550 °C for 2 h. Finally, the powder was ground and stored in a glass tube.

A powder Shimadzu XRD 7000 X-ray diffractometer (Kyoto, Japan) with a 2θ range of 5.0 to 80° was used to examine the crystal structure of nanocomposites. The samples were fixed on a glass holder. A Shimadzu 100 FTIR spectrometer was used to measure the FTIR spectra of samples. An environmental scanning electron microscope with an energy dispersive spectroscopy system (ESEM, Thermo Fisher with Oxford detector, Waltham, MA, USA) was used to examine structural morphology and elemental composition. The measurements of surface area and pore size were completed on the Quantachrome system (The NOVA A 4200e High- Speed). For the prepared samples, a Thermo Scientific Evolution 200 UV–vis spectrophotometer (Waltham, MA, USA) with a resolution of 0.1 nm was used to record the UV–vis spectra. The xenon lamp provides strong illumination from the UV to the near-IR region of the spectrum. An effective way to gain knowledge about material band structure and electron–hole recombination in photocatalysis is by the photoluminescence spectroscopy. A Cary Eclipse fluorescence spectrometer (Shimadzu, UK) was used to conduct photoluminescence spectroscopy. The excitation wavelength was selected to be 300 nm.

The inclusion methodology was used to assess the synthesized material’s hydrogen catalytic performance. Normally, 100 mL of distilled water was added without stirring after 10 mg of the nanocomposite sample had been combined with 1.0 g of NaBH_4_. The volume of hydrogen gas was measured using the water displacement method. Moreover, the measurements were carried out at 293, 303, 313 and 323 K.

## 3. Results and Discussion

**Figure 1:**
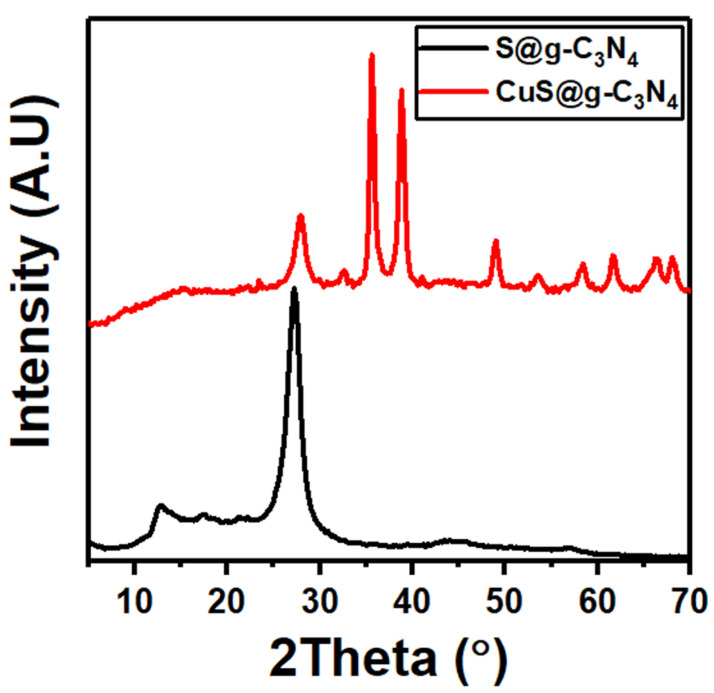
XRD diffraction patterns of S@g-C_3_N_4_ and CuS@g-C_3_N_4_ nanostructures.

The typical XRD patterns were used to identify the crystal structures of S@g-C_3_N_4_ and CuS@g-C_3_N_4_ nanocomposites. Figure 1 depicts the XRD spectra of the developed catalysts. The diffraction pattern of S@g-C_3_N_4_ presents a sharp peak at 27.2° and a weak peak at 13.1°, which corresponds to the (002) and (100) planes, respectively [47]. The sharp diffraction peak can be attributed to aromatic system interlayer stacking, whereas the weak diffraction peak can be attributed to aromatic in-plane structural packing. The diffraction pattern of CuS belongs to the hexagonal phase according to (PCPDF card No: 782121) and corresponds to (101), (006), (105), (110), (108), (116) and (118) planes with peak locations at 2θ = 27.8°, 32.6°, 38.8°, 49°, 53°, 58.4° and 66.4°, respectively [48]. The distinct and strong diffraction peaks provide evidence that the catalysts exhibit good crystallinity. The three main peaks (35.6°, 38.8° and 49°) were applied in the Scherer equation was used to determine the average crystallite grain size of CuS@g-C_3_N_4_ to be 10 nm [49,50]:

(1) $$D=\frac{0.9\lambda }{\beta cos\theta }$$

where *D* represents the typical crystallite grain size, λ represents the X-ray wavelength, and *β* represents full width at half maximum.

In graphitic carbon nitride, peaks centered at 13.1° and 27.2° are related to hydrogen bonding for sustaining intralayer long-range atomic order and van der Waals forces for managing interlayer periodic stacking along the c-axis [51]. The intensity of the peak located at 13.1^o^ was reduced after the growth of the CuS nanosheet demonstrating that the long-range order of the in-plane structural packing in the g-C_3_N_4_ sheets has been much reduced because of hydrogen bond breaking in the intralayer framework [52]. The specific peak of g-C_3_N_4_ located at 27.2° is shifted to 27.8°, which is due to the addition of the CuS group on g-C_3_N_4_ nanosheets. The interplanar distance decreased from 0.328 to 0.319 nm which facilitated charge carrier separation and promoted H_2_ generation. In addition, the C–N sheet structure of S@g-C_3_N_4_ moiety of CuS@g-C_3_N_4_ is slightly changed, resulting in a drop in the intensity peak of CuS@g-C_3_N_4_ compared to S@gC_3_N_4_ [53,54]. Moreover, the periodic stacking of the layers may be disturbed by the hydrogen bonding-free layers [51].

**Figure 2:**
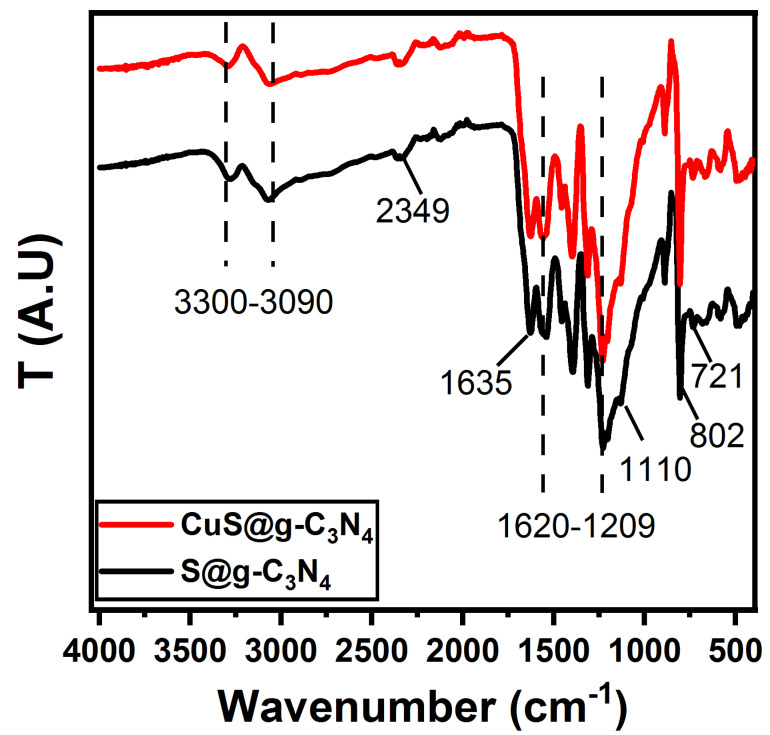
FTIR spectra of S@g-C_3_N_4_ and CuS@g-C_3_N_4_ nanostructures.

The Fourier transform infrared spectroscopy (FTIR) spectra of S@g-C_3_N_4_ and CuS@g-C_3_N_4_ nanosheet are presented in Figure 2. The existence of the S–C bond at 721 cm^−1^ in S@gC_3_N_4_ indicated that sulfur was successfully incorporated into the g-C_3_N_4_ structure [55]. Due to the significantly larger ionic radius of copper (approximately 145 picometers) and sulfur (approximately 180 picometers) in comparison to carbon and nitrogen (measuring at 70 and 65 picometers, respectively), it is unlikely that substitution doping will take place. Furthermore, it has been established that g-C_3_N_4_ is a compound held together by covalent bonds. The doping of Cu^+^ and S^+^ as an ion state in a substitutional site was found to be unfeasible. Furthermore, according to reference [56], the maximum interplanar distance of nitride pores is 0.71 nm, which is sufficient to accommodate Cu+ and S+. This finding verifies that interstitial doping took place, while substitution doping was not present [57]. The absorption bands at 802 and 1209–1620 cm^−1^ are attributed to aromatic in-plane structural packing C=C/C=N/C-N bonds [58]. The peaks at 1635 cm^−1^ and 1110 cm^−1^ were attributed to the hydroxyl groups on the surface of hydrated oxide and thioacetamide on the surface of CuS, respectively [59]. The band at 2349 cm^−1^ is assigned to adsorbed CO_2_ while the bands located at 3090–3300 cm^−1^ are attributed to NH and OH groups [60,61].

**Figure 3:**
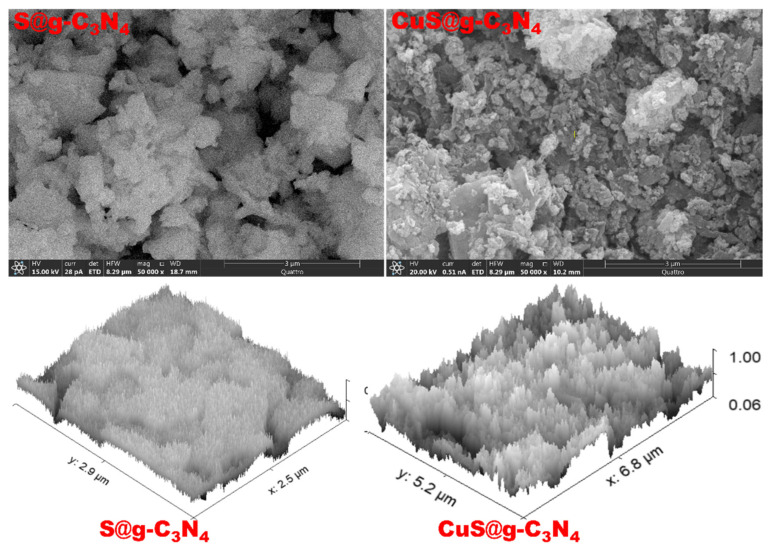
ESEM images of S@g-C_3_N_4_ and CuS@g-C_3_N_4_ nanostructures.

ESEM electron spectroscopy was used to investigate the structure of S@g-C_3_N_4_ and CuS@g-C_3_N_4_ nanostructures. Figure 3 demonstrates that S@g-C_3_N_4_ exhibits the described layered sheet structure for g-C_3_N_4_ materials. In Figure 3, CuS@g-C_3_N_4_ demonstrates that the sheet materials have been fragmented throughout the growth process, exposing additional edge sites. Therefore, the CuS@g-C_3_N_4_ nanosheet is expected to possess high surface area and porosity. The 3D surface plot was also provided in Figure 3 for S@g-C_3_N_4_ and CuS@g-C_3_N_4_. The obtained plots confirm the sheet morphology of S@g-C_3_N_4_ and fragmented flakes for CuS@g-C_3_N_4_.

Surprisingly, XRD analysis and ESEM microscope images revealed a layered structure of CuS@g-C_3_N_4_ nanocomposite and supported the formation of interlayers of CuS between g-C_3_N_4_ nanosheets. Moreover, this leads to a decrease in the interplanar distance and thus delays the electron–hole recombination.

**Figure 4:**
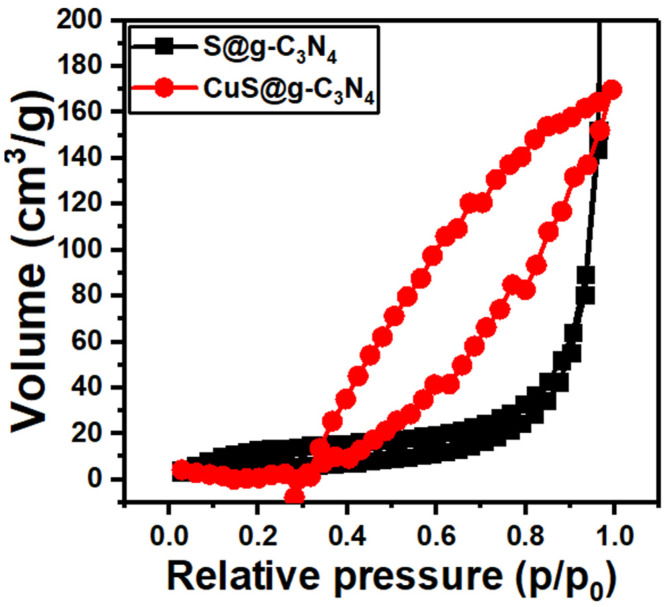
N_2_ isotherm for S@g-C_3_N_4_ and CuS@g-C_3_N_4_ nanostructures.

The surface area of the S@g-C_3_N_4_ and CuS-g-C_3_N_4_ nanocomposite samples was determined using the N_2_ adsorption–desorption isotherm plotted in Figure 4. The samples showed type IV isotherm without saturation, indicating mesoporous architecture. The data of BET revealed a surface area of 40 and 55 m^2^/g for the samples S@g-C_3_N_4_ and CuS-g-C_3_N_4_. Meanwhile, the Barett-Joyner-Halenda (BJH) pore volume analysis showed 0.24 cm^3^ for S@g-C_3_N_4_ and 0.34 cm^3^ for CuS-g-C_3_N_4_. This shows an increase in pore volume after the growth of CuS nanoparticles. Therefore, the polycondensation process helped to increase the porosity of nanostructures and thus enhance the catalytic performance of these materials as the number of active sites is increased [62,63].

**Figure 5:**
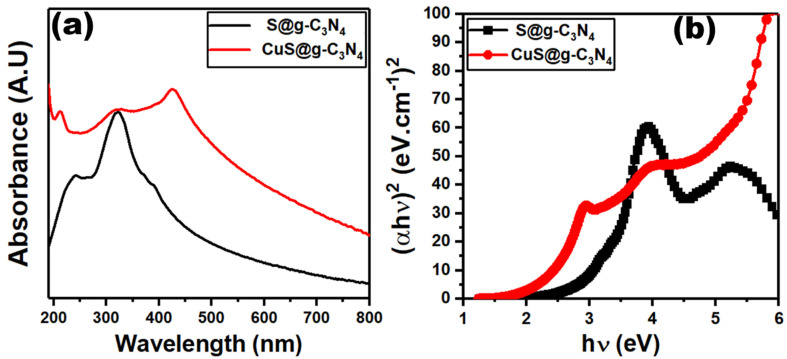
Plots of (**a**) absorbance vs. wavelength and (**b**) (ahν)^2^ vs. photon energy for S@g-C_3_N_4_ and CuS@g-C_3_N_4_ nanostructures.

The capacity of a material to absorb light in the visible, near-UV, and near-infrared regions of the electromagnetic spectrum is investigated using the UV–vis spectrophotometry analysis. The UV–vis absorption spectra of S@g-C_3_N_4_ and CuS@g-C_3_N_4_ nanostructures are displayed in Figure 5a. The spectrum of S@g-C_3_N_4_ showed a strong peak at 322 nm, which comes because of n→π* electronic transitions [64]. This peak is weakened after the growth of CuS at g-C_3_N_4_. Accordingly, the growth of CuS@g-C_3_N_4_ produces a change in the electronic structure of g-C_3_N_4_ and affects the photo-induced electron–hole generation.

The optical bandgap investigation gives more information about the electronic structure of materials. Photon absorption (*αhv*) and optical bandgap (*E_opt_*) have the following mathematical relationship [65,66]:

(2) $$\alpha hv=A{\left(hv-E_{opt}\right)}^{n}$$

where *A* is a constant and *n* = 0.5 for direct allowed transitions. The intercept of straight lines at (αhν)^2^ = 0 for the graphs shown in Figure 5b gives the values of the optical band gap. Therefore, the estimated band gaps of S@g-C_3_N_4_ and CuS@g-C_3_N_4_ nanostructures are 2.6 and 2.3 eV. The development of new energy levels or changes in the electronic structure of g-C_3_N_4_ accounts for bandgap reduction [67,68]. The up-shift of the valence band (VB) and the downshift of the conduction band (CB) cause the bandgap to shrink in the CuS@g-C_3_N_4_ sample [69]. The intercalation of CuS molecules between g-C_3_N_4_ interlayers bridges the layers that reduce the electronic localization and spread the p-conjugated system [70].

**Figure 6:**
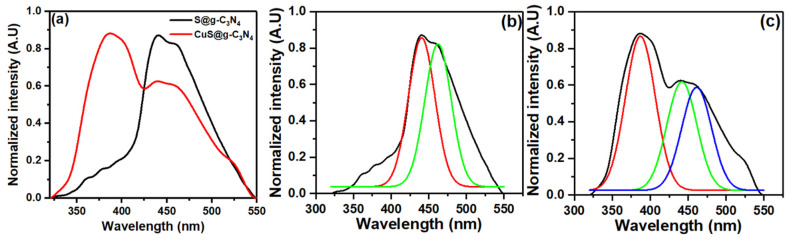
Photoluminescence scans for (**a**) S@g-C_3_N_4_ and CuS@g-C_3_N_4_ nanostructures, (**b**) deconvoluted spectrum of S@g-C_3_N_4_ and (**c**) deconvoluted spectrum of CuS@g-C_3_N_4_.

The photoluminescence (PL) analysis of the catalyst gives information about charge separation dynamics and the electron–hole recombination rates [71]. Accordingly, the emission spectra of S@g-C_3_N_4_ and CuS@g-C_3_N_4_ are displayed in Figure 6. The two samples showed a PL emission at 441 nm, which correlated with electron–hole pair recombination. Meanwhile, the intensity of this band decreased after the growth of CuS. This leads to high separation of photo-induced electron–hole pairs [72]. This finding reveals that after CuS at g-C_3_N_4_, the recombination rates were dramatically lowered. Thus, the catalytic performance of this sample is expected to be improved.

For the CuS@g-C_3_N_4_ sample, an additional emission peak located at 387 nm was observed in Figure 6c. This emission peak is attributed to copper sulfide nanoflakes and this result agrees with the literature [73].

**Figure 7:**
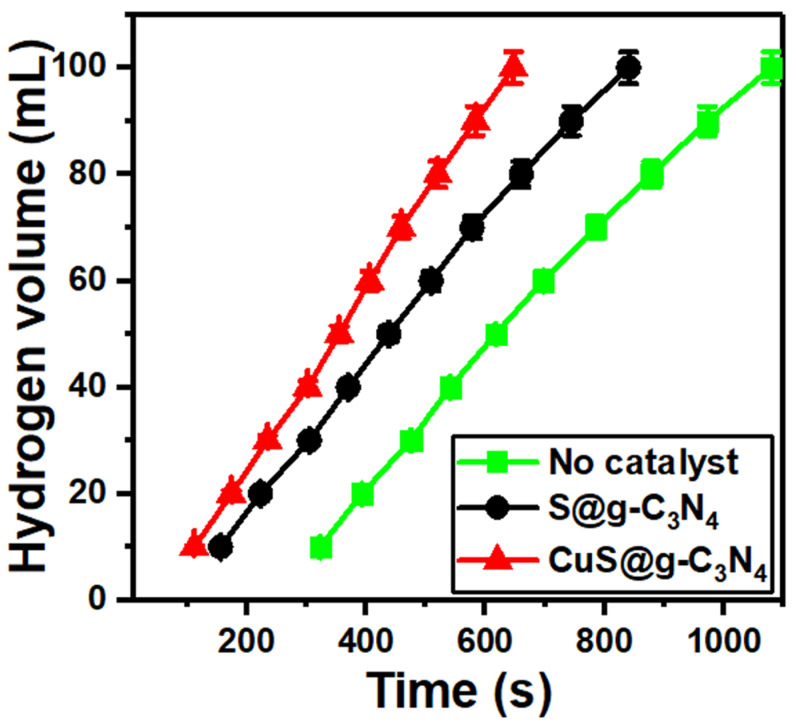
Hydrogen generation from NaBH_4_ for S@g-C_3_N_4_ and CuS@g-C_3_N_4_ nanostructures at 293 K.

We studied the hydrogen generation from 1.0 g of NaBH_4_ and added the catalysts, which are 0.01 g of S/g-C_3_N_4_, and 0.01 g of CuS/g-C_3_N_4_. Figure 7 represents the hydrogen generation volume against the time for NaBH_4_ (no catalyst), S@g-C_3_N_4_ and CuS@g-C_3_N_4_; the experiment completed at 293 K. It is shown that the addition of only 0.01 g catalyst accelerates the hydrogen production. The highest hydrogen production was achieved for CuS@g-C_3_N_4_. This comes because of more active sites located at the surface of CuS@g-C_3_N_4_ as explained by surface area and pore size analysis. Understanding the active sites will assist in the design and manufacturing of catalysts with increased activity, selectivity, and stability. Nanocatalysts have active regions of many catalytic processes only include a small number of atoms or minority species. When compared to the atoms in the bulk, these various surface atoms have distinct chemical environments, which may also cause variations in charge redistribution at the interface [74]. For many catalytic applications, corners and edges are now generally acknowledged to be more effective active sites [75]. The morphological structure of g-C_3_N_4_ has a significant impact on its performance. There are few active surface sites in conventional g-C_3_N_4_ because of its layered bulk structure. Therefore, the number of active surface sites can be increased by activating the g-C_3_N_4_ surface. Further, the addition of an active material capable of precisely adsorbing the reaction substrates increases the active sites of g-C_3_N_4_. Due to the increased exposure of active sites, the 2D structure of photocatalysts offers enormous promise. An efficient method to lower the activation barrier for catalytic processes competing with the recombination of photogenerated carriers is to add a supportive cocatalyst to enlarge the active site [76].

**Figure 8:**
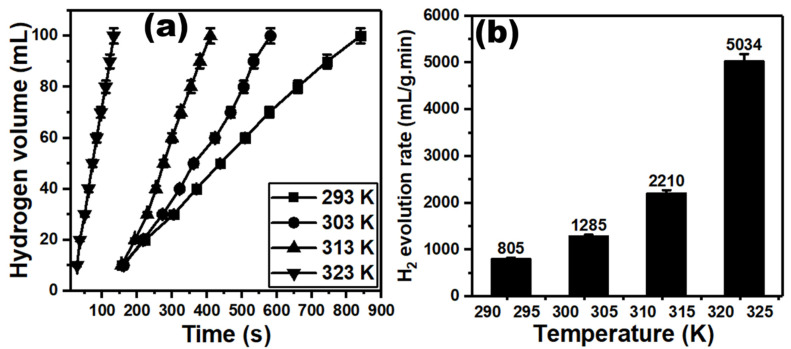
Plots of (**a**) hydrogen volume versus time and (**b**) hydrogen evolution rate versus the temperature for S@g-C_3_N_4_.

The hydrogen evolution of S@g-C_3_N_4_ and CuS@g-C_3_N_4_ was measured at different temperatures (239, 303, 313, and 323 K). Figure 8a,b represents the increase in the hydrogen production of S@g-C_3_N_4_ when the temperatures increased from 293 to 323 K. The hydrogen generation rate (*K*) is connected to the volume of hydrogen (*V*), the mass of the catalyst (*m_cat_*) and time (*t*) through the following equation [77,78];

(3) $$K=\frac{V}{t.m_{cat}}$$

**Figure 9:**
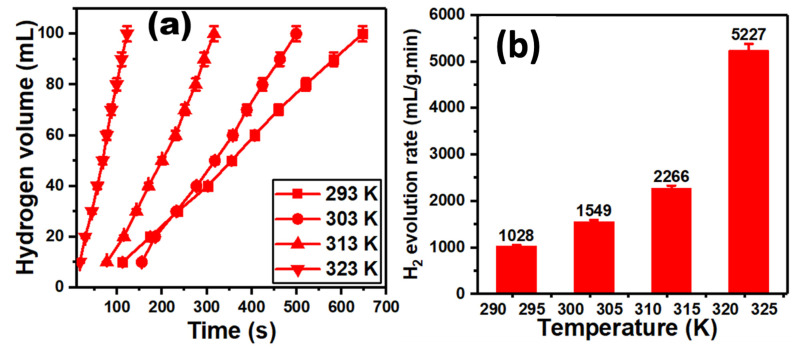
Plots of (**a**) hydrogen volume versus time and (**b**) hydrogen evolution rate versus the temperature for CuS@g-C_3_N_4_.

Figure 8b represents the value of the highest hydrogen evolution rate, which is 5034 mL/g·min at 323 K. The lowest value is 805 mL/g·min at 293 K. Moreover, the data of hydrogen production for CuS@g-C_3_N_4_ were displayed in Figure 9a. The increase in temperature improves hydrogen production. Further, the second catalyst CuS@g-C_3_N_4_ highest hydrogen evolution rate is 5227 mL/g·min at 323 K, as shown in Figure 9b.

**Figure 10:**
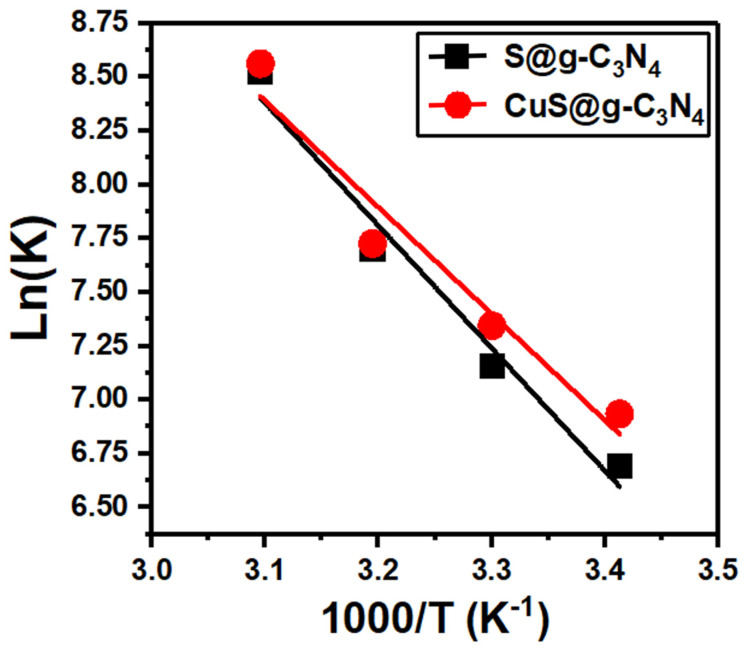
Plots of ln(K) vs. 1000/T for S@g-C_3_N_4_ and CuS@g-C_3_N_4_ nanostructures.

The data of hydrogen generation rate vs temperature allow for the estimation of activation energy (*E_a_*). In this context, the following Arrhenius relation connects the activation energy for NaBH_4_ hydrolysis to the temperature (*T*) [78,79]:

(4) $$\ln\left(K\right)=\ln\left(A\right)-\frac{E_{a}}{RT}$$

where is an exponential factor and *R* defines the gas constant (8.314 kJ K^−1^ mol^−1^). The slope of straight lines shown in Figure 10 helps with *E_a_* calculations. The apparent activation energy was determined for S@g-C_3_N_4_ and CuS@g-C_3_N_4_, which showed values of 47.33 ± 0.02 and 41.15 ± 0.02 KJ/mol. Moreover, the activation energies of S@g-C_3_N_4_ and CuS@g-C_3_N_4_ nanostructures are lower than that for Co-P/CNTs-Ni foam catalyst [80], Co–Mo–B/C [81], Co_3_O_4_@TiO_2_-g-C_3_N_4_ [82], Co@TiO_2_ [83] and CoB/Ag–TiO_2_ [84] as seen in Table 1.

A comparison of hydrogen generation rate and activation energy for our nanocatalyst and other materials is listed in Table 1. The data recorded in this table indicated that the prepared CuS@g-C_3_N_4_ catalyst is superior to other materials.

## 4. Conclusions

The nanocomposites of S@g-C_3_N_4_ and CuS@g-C_3_N_4_ catalysts were prepared via the polycondensation process. XRD and FTIR analysis confirmed the structural transformation of S@g-C_3_N_4_ and CuS@g-C_3_N_4_. ESEM images of S@g-C_3_N_4_ exhibited the described layered sheet structure for g-C_3_N_4_ materials and CuS@g-C_3_N_4_ demonstrated that the sheet materials were fragmented throughout the growth process. BET data revealed a surface area of 40 and 55 m^2^/g for the samples S@g-C_3_N_4_ and CuS-g-C_3_N_4_. Meanwhile, the BJH pore volume analysis showed 0.24 cm^3^ for S@g-C_3_N_4_ and 0.34 cm^3^ for CuS-g-C_3_N_4_. UV–Vis absorption measurements showed that the estimated band gaps of S@g-C_3_N_4_ and CuS@g-C_3_N_4_ nanostructures are 2.6 and 2.3 eV. The two samples showed a PL emission at 430–480 nm, with the intensity of this band decreasing after the growth of CuS. The data of hydrogen evolution showed that the sample CuS@g-C_3_N_4_ has high generation rates and lower activation energy 41.15 ± 0.02 KJ/mol. These findings approve the importance of the prepared CuS@g-C_3_N_4_ nanostructures for hydrogen production from NaBH_4_.

## Figures and Tables

**Table 1:** Catalysts for hydrogen release through hydrolysis of NaBH_4_.

Catalyst	Form	Hydrogen Evolution Rate (mL/g·min)	Activation Energy (KJ/mol)	Ref.
Co-P/CNTs-Ni	Foam	2640	47.27	[79]
Co–Mo–B/C	Powder	1280.8	51.0	[80]
Co_3_O_4_@TiO_2_-g-C_3_N_4_	Powder	1200	58.0	[81]
Co@TiO_2_(P25)	Powder	660	45.2	[82]
CoB/Ag–TiO_2_	Powder	393	44.0	[83]
CuS@g-C_3_N_4_	Powder	5227	41.15 ± 0.02	This study

## Data Availability

The data that support the findings of this study are available from the author upon reasonable request.
